# Supplementary figures and images for: The illusion of information adequacy
Source: PLoS One. 2024 Oct 9;19(10):e0310216. doi: 10.1371/journal.pone.0310216 (PMC11463766; doi:10.1371/journal.pone.0310216)

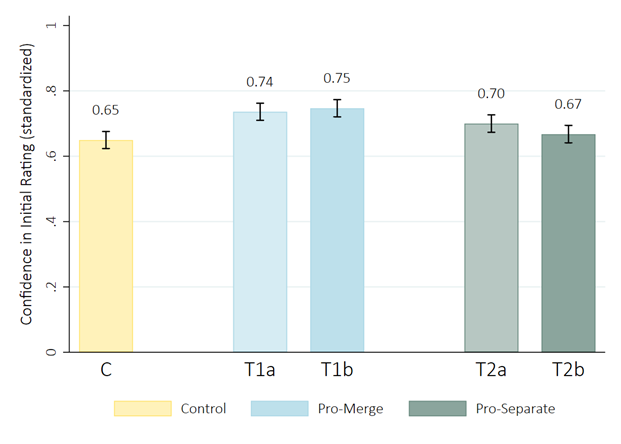

Supplement: S1 Fig — Comparison of the four different treatment groups versus the control group in their initial responses to the question, “How confident are you that your recommendation is the smartest action for the school board to take?”. (TIF) [file pone.0310216.s001.tif]

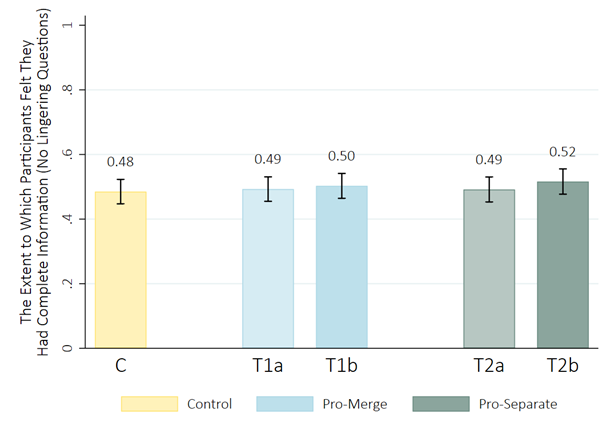

Supplement: S2 Fig — Panel A. Perceived Key Details Received by Condition and 95% CIs. Comparison of the four different treatment groups versus the control group in their responses to the question, “Sometimes people are satisfied that they have all the information that they need to make a decision. Other times, they feel that they need more information. After reading the article, to what extent do you feel that you understand enough of the key details of the situation to make a good decision?”, Panel B. Perceived Lack of Lingering Question by Condition and 95% CIs. Comparison of the four different treatment groups versus the control group in their responses to the question, “To what extent do you feel as though you still have questions about important details of Prairie View’s situation?”. (ZIP) [file pone.0310216.s002.zip › S2B_Fig.tif]

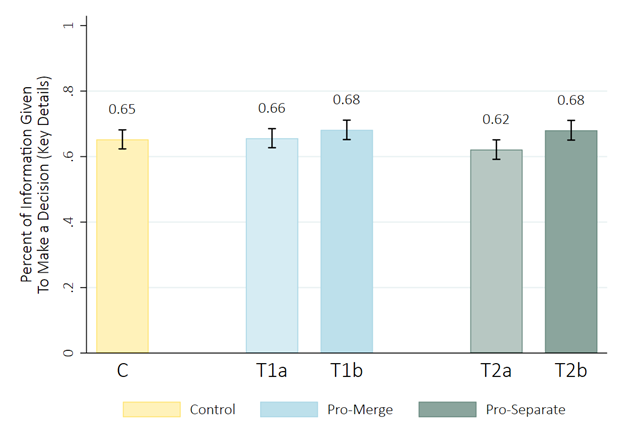

Supplement: S2 Fig — Panel A. Perceived Key Details Received by Condition and 95% CIs. Comparison of the four different treatment groups versus the control group in their responses to the question, “Sometimes people are satisfied that they have all the information that they need to make a decision. Other times, they feel that they need more information. After reading the article, to what extent do you feel that you understand enough of the key details of the situation to make a good decision?”, Panel B. Perceived Lack of Lingering Question by Condition and 95% CIs. Comparison of the four different treatment groups versus the control group in their responses to the question, “To what extent do you feel as though you still have questions about important details of Prairie View’s situation?”. (ZIP) [file pone.0310216.s002.zip › S2A_Fig.tif]
