# Supplementary material for: The illusion of information adequacy
Source: PLoS One. 2024 Oct 9;19(10):e0310216. doi: 10.1371/journal.pone.0310216 (PMC11463766; doi:10.1371/journal.pone.0310216)
Supplement: Attachment — Submitted filename: InformationAdequacy_ResponseToReviews(7-12-24).docx [file pone.0310216.s003.docx]

| **Comments** | **Actions Taken** |
| --- | --- |
| Please ensure that your manuscript meets PLOS ONE's style requirements, including those for file naming. | Thank you for the recommended templates. We believe we have adhered to all style requirements, but please let us know if we have missed anything. |
| We note that the grant information you provided in the ‘Funding Information’ and ‘Financial Disclosure’ sections do not match. | We assume that you are referring to sections of the submission portal. In the final screen, “Manuscript Data,” we can see a section for “Funding Information.” That correctly reads that funds came from JHU’s School of Education—i.e., these were start-up funds provided to the first author. Although we can see the “Financial Disclosure” not in the .pdf file of our submission, we cannot navigate back to edit that section—not sure why? Regardless, that statement is also true that there was no specific grant that funded this work. Please also note that there are no grant numbers for start-up funds. Perhaps it would be best for you to decide how you would like us to communicate this funding arrangement and let us know how we can navigate to the “Financial Disclosure” section if that requires editing? |
| When completing the data availability statement of the submission form, you indicated that you will make your data available on acceptance. | The data set and code are now available at: https://osf.io/k37dg/ |
| Please include your full ethics statement in the ‘Methods’ section of your manuscript file. In your statement, please include the full name of the IRB or ethics committee who approved or waived your study, as well as whether or not you obtained informed written or verbal consent. | We state that, “The procedures for both studies were approved by the **Johns Hopkins University Human Subjects Committee**, and all participants provided **written informed consent** prior to participation by clicking a button that indicated their consent.” |
| Please include captions for your Supporting Information files at the end of your manuscript, and update any in-text citations to match accordingly. | We have included notes for each of the 3 figures in the Supporting Information. |
| The authors claim that their study revolves around a "local, hypothetical scenario—for which they could not have held previous beliefs." I do not share the assumption that participants read the scenario without any preexisting biases. | We have reworded this sentence to soften the language and make clear that we are *not* suggesting that the participants read the scenario free from any preexisting biases:  “We asked participants about a local, fictitious scenario—for which they were unlikely to hold strong prior opinions given the hypothetical nature of the scenario.” |
| In the case of equivalence t-tests, the authors should mention the exact name of the test they used, since multiple implementations are available. | We clarify that we are following the approach of Lakens et al:  Lakens D, Scheel AM, Isager PM. Equivalence testing for psychological research: A tutorial. Advances in Methods and Practices in Psychological Science. 2018;1(2):259-69. doi: 10.1177/2515245918770963. |
| It is unclear from the manuscript why some treatment groups completed different measures than others. This is rather confusingly presented in Figure 1 and the authors do not explain why the names of some measures are italicized. | In the figure caption, we have clarified that some of the questions were likely to bias respondents and so the second randomization of treatment participants allowed us to assess the impact of this potential bias. We have also explained that the italicized words correspond to their use in the measures section. |
| While revising your submission, please upload your figure files to the Preflight Analysis and Conversion Engine (PACE) digital diagnostic tool, [https://pacev2.apexcovantage.com/](https://nam02.safelinks.protection.outlook.com/?url=https%3A%2F%2Fpacev2.apexcovantage.com%2F&data=05%7C02%7Cgehlbach%40jhu.edu%7C524b6ee5d4084595b39b08dc8e88e12f%7C9fa4f438b1e6473b803f86f8aedf0dec%7C0%7C0%7C638541967173465698%7CUnknown%7CTWFpbGZsb3d8eyJWIjoiMC4wLjAwMDAiLCJQIjoiV2luMzIiLCJBTiI6Ik1haWwiLCJXVCI6Mn0%3D%7C0%7C%7C%7C&sdata=yujvfUYww6gPCZyC7IKEwkWUWzVu%2BcMTA94H2h3w%2BF4%3D&reserved=0). | This has been done. |
